# Exposure to clinical stressors during NICU admission in preterm infants

**DOI:** 10.1007/s00431-025-06018-7

**Published:** 2025-02-13

**Authors:** Naomi J. Meesters, Gerbrich E. van den Bosch, Maria Luisa Tataranno, Chris H. P. van den Akker, Christ-jan van Ganzewinkel, Judith A. ten Barge, Frank A. B. A. Schuerman, Henriette van Zanten, Willem P. de Boode, Marlou M. A. Raets, Peter H. Dijk, Joost van Rosmalen, Marijn J. Vermeulen, Wes Onland, Lotte Haverman, Irwin K. M. Reiss, Anton H. van Kaam, Manon Benders, Monique van Dijk, Sinno H. P. Simons, the HIPPO study group

**Affiliations:** 1 Division of Neonatology, Department of Neonatal and Pediatric Intensive Care, Erasmus MC –Sophia Children’s Hospital, Wytemaweg 80, 3015 CN Rotterdam, The Netherlands; 2 Department of Neonatology, University Medical Center Utrecht, Brain Center, Utrecht University, Utrecht, the Netherlands; 3 Department of Neonatology, Emma Children’s Hospital, Amsterdam UMC, University of Amsterdam, Amsterdam Reproduction & Development Research Institute, Amsterdam, the Netherlands; 4 Department of Neonatology, Máxima Medical Center, Veldhoven, The Netherlands; 5 Department of Neonatology, Isala Women and Children’s Hospital, Zwolle, The Netherlands; 6 Division of Neonatology, Department of Pediatrics, Leiden University Medical Center, Leiden, the Netherlands; 7 Division of Neonatology, Department of Paediatrics, Radboud University Medical Center, Radboud Institute for Health Sciences, Amalia Children’s Hospital, Nijmegen, The Netherlands; 8 Division of Neonatology, Department of Pediatrics, Maastricht University Medical Center+, MosaKids Children’s Hospital, Maastricht, The Netherlands; 9 Department of Neonatology, Beatrix Children’s Hospital, University Medical Center Groningen, University of Groningen, Groningen, The Netherlands; 10 Department of Biostatistics, Erasmus MC, Rotterdam, The Netherlands; 11 Department of Epidemiology, Erasmus MC, Rotterdam, The Netherlands; 12 Care4Neo, Neonatal Patient and Parent Organisation, Rotterdam, The Netherlands; 13 Neonatology Network Netherlands, Amsterdam UMC, University of Amsterdam, Amsterdam, The Netherlands; 14 Child and Adolescent Psychiatry & Psychosocial Care, Emma Children’s Hospital, Amsterdam UMC, University of Amsterdam, Amsterdam Reproduction and Development, Amsterdam Public Health, Amsterdam, The Netherlands

**Keywords:** Prematurity, Stress, Pain, NICU

## Abstract

This study aims to quantify stress exposure related to clinical stressors in preterm infants during NICU admission and identify risk factors for high stress exposure. In this national cohort study, preterm infants (gestational age < 29 weeks) were prospectively followed during the first 28 days of their admission to one of the 10 NICUs in the Netherlands. The NeO-stress score, consisting of 38 clinical stressors graded with a severity index, was applied to describe stress exposure. We assessed the impact of infant characteristics at birth and postnatal age on NeO-stress scores using linear mixed modelling. In total, 446 infants were included with a median gestational age of 27^+2^ weeks (*IQR* 26^+2^–28^+2^). The median NeO-stress score per day was 61 (*IQR* 39–87) and highest (74, *IQR* 52–101) on the day of admission. Nasal/oral (37%) and endotracheal (14%) suctioning were key contributors to the cumulative NeO-stress scores. Linear mixed modelling showed that lower gestational age (*B* = -0.69, 95% *CI* − 0.94–0.44, *p* < 0.001), no antenatal administration of corticosteroids (*B* = 13.2, 95% *CI* 3.2–23.1, *p* = 0.010) and lower 5-min Apgar score (*B* = − 1.6, 95% *CI* − 3.0–0.25, *p* = 0.02) were significantly related with higher daily NeO-stress scores. Our model predicts that the NeO-stress score increases over time for the youngest infants.

*Conclusion*: Stress exposure in preterm infants during NICU admission varies over time with infants with the lowest gestational age at risk for experiencing the highest levels of stress throughout NICU admission. This highlights the importance stress reduction and provides opportunities for future interventions aimed at reducing stress exposure.

**Table:** 

**What is Known:** *• Preterm birth and admission to a Neonatal Intensive Care Unit is very stressful.* *• High stress exposure in neonatal life is associated with adverse long term outcome.*	
**What is New:** *• Stress exposure is highest in infants with the youngest gestational ages where it remains high or even increases during the first month of life.* *• Lower gestational age, no antenatal administration of corticosteroids and lower 5-min Apgar score were significantly related with higher daily NeO-stress scores.*

**What is Known:**

*• Preterm birth and admission to a Neonatal Intensive Care Unit is very stressful.*

*• High stress exposure in neonatal life is associated with adverse long term outcome.*

**What is New:**

*• Stress exposure is highest in infants with the youngest gestational ages where it remains high or even increases during the first month of life.*

*• Lower gestational age, no antenatal administration of corticosteroids and lower 5-min Apgar score were significantly related with higher daily NeO-stress scores.*

## Introduction

Extremely preterm born infants receive long lasting treatment in a Neonatal Intensive Care Unit (NICU). Many of the available treatment options are inevitably stressful for the infants and induce acute and prolonged pain and stress, e.g. skin-breaking procedures and mechanical ventilation [1, 2]. While NICU treatment aims to increase survival after preterm birth, the exposure to pain and stress itself can negatively impact long-term developmental outcome [3–9].

Defining “stress” in preterm born infants admitted to a NICU is difficult. Lu et al. recently proposed a framework that identifies five consecutive basic elements of stress: stimulus, stressor, stress, stress response and stress effect [10]. Stressful stimuli may become stressors if they directly challenge the homeostasis, referred to as stress. Stress activates the hypothalamic–pituitary–adrenal (HPA) axis, which results in a stress response. This response can ultimately lead to physical and psychological disorders, referred to as the stress effect [10]. Potential stressors related to NICU environment can be categorized by their origin: clinical stressors (e.g. NICU treatment), physical stressors (e.g. noise) and psychological stressors (e.g. lack of parental presence) [9]. In this study, we will focus on stress induced by clinical NICU stressors, which we refer to as ‘stress exposure’.

Previous studies showed that a higher stress exposure in preterm infants is associated with adverse neurobehavioral outcome at term equivalent age and beyond [7, 8]. A first step to protect infants from these negative consequences would be to reduce the number of clinical stressors as much as possible. Secondarily, we should reduce the level of stress associated with each stressor, for example by applying skin-to-skin care [11, 12].

We report a prospective national cohort study with the primary aim to quantify stress exposure in preterm infants (born at a gestational age less than 29 weeks) during the first 28 days of their NICU admission and identify the most important clinical stressors. As secondary aims, we identified infant characteristics at birth that may predict the infant’s daily stress exposure and determined differences in stress exposure across all Dutch level III/IV NICUs.

## Materials and methods

### Design

This national multicentre observational cohort study (Happiness for the Improvement of Premature and Parental outcome — HIPPO study, Dutch trial register NL8939) followed preterm infants prospectively during their NICU admission. Data collection for all eligible patients started immediately after birth (day 1) and continued until the 28th day of life, since most stressors occur during this period. Moreover, most children are transferred to a post-NICU centre from a gestational age of 30 weeks onwards. Parents were informed about this study as early as possible. Data collection stopped before day 28 if parental consent was not granted or after death or NICU discharge.

### Patients and setting

All preterm infants born at a gestational age below 29 weeks admitted to one of all 10 level III/IV NICUs across the Netherlands were eligible for inclusion. This cut-off for gestational age was chosen since infants may leave the NICU from a postmenstrual age of 30 weeks onwards. With an average of 600 to 700 preterm infants with a gestational age below 29 weeks admitted to Dutch NICUs annually, we aimed to include at least 400 infants.

Since NICUs started patient enrolment at different times, the total inclusion period spanned from July 1, 2020 until March 1, 2022, with each NICU having a 1-year inclusion period. Infants were recruited by the local research team and excluded only if parents could not read the written information (Dutch, English, Turkish, Arabic or Polish).

The medical ethical committee of the Erasmus MC waived the requirement for approval under Dutch Law on research with humans (MEC-2019–0574), as the study was deemed non-interventional and did not subject infants to procedures or additional behavioural rules. The medical ethical committees of the other participating NICUs subsequently adhered to this decision.

### Data collection

All data were entered in Castor® Electronic Data Capturing system (Amsterdam, the Netherlands) by the local research teams, an online database that complies with Good Clinical Practice (GCP) guidelines.

### NeO-stress score

We developed the NeO-stress score in preparation of the current study to gain insight into a preterm infant’s stress exposure and estimate the cumulative amount of stress [13]. Different instruments have been developed previously to quantify stress exposure. However, none of these instruments have been validated for NICU populations outside the US or Australia, and regarding face validity, Dutch NICU nurses and physicians were of the opinion that existing instruments such as the “Neonatal Infant Stressor Scale” (NISS), “Procedural Load Index” (PLI) and “Accumulated Pain/Stressor Scale” (APSS) [1, 14, 15] did not match their current clinical practice. We therefore developed an instrument to quantify very preterm infants’ daily cumulative stress level during the first 28 days of life. In brief, NICU professionals across the Netherlands scored the relevance and comprehensibility of 77 potentially stressful items as well as the comprehensiveness of the item list. Calculating the content validity per item (CVI-I) resulted in a list of 38 relevant items, of which 34 had a CVI-I if 0.78 or higher. One of these items was split into two items, and three items were added to improve comprehensiveness. In a second round, the participants rated the stressfulness of the items from 0 (not stressful) to 10 (extremely stressful). A stressfulness index — representing the median score — was calculated for each included item [13] (SI, see supplement 1). The NeO-stress score is calculated per day by multiplying the number of times each stressor occurred by the *SI* (NeO-stress = *N*_item1_ × *SI*_item1_ + *N*_item2_ × SI_item2_ + etc.) [13]. The stressors were registered prospectively in a study diary by the health caregivers or retrieved from the electronic health record system.

Since “Continuous Positive Airway Pressure (CPAP) respiratory support” accidently did not receive a *SI* in our initial instrument development study, we calculated the median *SI* for non-invasive respiratory support based on the rating of 12 nurses and 13 physicians from all Dutch NICUs and added this item to the NeO-stress score (non-invasive respiratory support for 24 h = *SI* 6).

### Infant characteristics

Infant characteristics included gestational age, birthweight, sex, small for gestational age (< 10th percentile, Fenton 2013 growth charts[16]), inborn/outborn, singleton/twin/triplet and 5-min Apgar score. Maternal characteristics (antenatal administration of corticosteroids, magnesium sulfate (MgSO_4_) and smoking during pregnancy) were retrieved from patient records. We recorded the type of NICU admission: single beds (2 NICUs) or open bay (8 NICUs).

### Statistical analysis

Infants’ characteristics are presented as median and interquartile range (*IQR*) for continuous non-normally distributed variables and as the number of participants (percentage) for categorical variables.

### Part A — quantification of stress exposure during NICU admission

We calculated the cumulative NeO-stress scores per day per infant. In order to determine the contribution of each individual stressor, for each item we multiplied the number of times the item occurred during the first 28 days by the *SI* (*N*_item_ × *SI*_item_) and divided this score by the total cumulative NeO-stress score during the admission days (maximum 28 days).

### Part B — factors associated with the level of stress exposure

#### B.1 Infant characteristics associated with the level of stress exposure

- Linear mixed-effects modelling was applied with the NeO-stress scores per day as the outcome variable and infant and maternal characteristics as predictor variables based on their judged clinical relevance (Model 1). A random intercept and slope per infant were included in the model together with an AR(1) covariance matrix to account for within-infant correlations. Infants with missing data were excluded since these data might be missing not at random.
- The interaction term of postnatal age and gestational age was added in Model 2 in order to examine whether the change in stress exposure over time varied between infants of different gestational ages. Possible nonlinear effects of postnatal age and gestational age were assessed by including natural cubic splines of these variables. Because the spline variables did not improve the model fit, only linear terms of postnatal age and gestational age were retained in the final model.

#### B.2 Differences in stress exposure between NICUs

The unit of admission and the unit type (open bay or single bed unit) were separately added as predictor variables to the final model in Models 3 and 4, respectively. This enabled us to compare the differences in NeO-stress scores, taking into account the possible differences between units with respect to the infant characteristics included in the model.

A *p*-value < 0.05 was considered statistically significant in all analyses. Data were analysed using IBM SPSS Statistics for Windows, version 27.0. Armonk, NY and R version 4.2.1 with the nlme package (version 3.1–157) to estimate linear mixed models.

## Results

### Infant characteristics

**Fig. 1:**
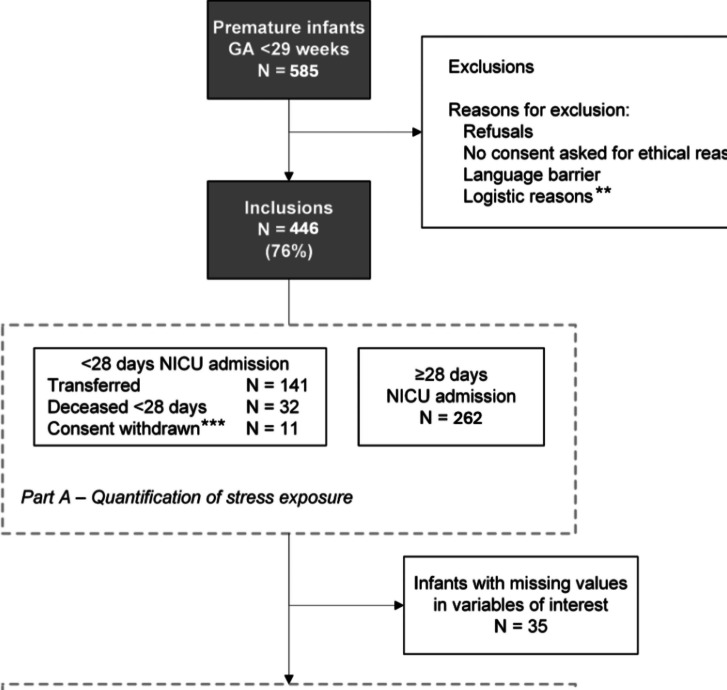
Inclusion flowchart. * I.e. in case of palliative care or severe illness of the mother. ** I.e. no daytime presence of the parents. *** Permission to analyse the collected data until consent withdrawal

During the inclusion period, 585 infants with a gestational age less than 29 weeks were born in the 10 NICUs, of whom 446 (76%) were included in this study (Fig. 1). A median of 40 infants (range 29 to 101) were included per NICU. Table 1 presents an overview of the baseline infant characteristics for the 446 preterm infants included in this study.

**Table 1:** Infant characteristics (*N* = 446)

Variable	*N*	*n* (%)	Median (*IQR*)
Gestational age (weeks^+days^)	446		27^+2^ (26^+2^ to 28^+2^)
23^+6^ weeks		3 (1%)	
24^+0–6^ weeks		31 (7%)	
25^+0–6^ weeks		58 (13%)	
26^+0–6^ weeks		91 (20%)	
27^+0–6^ weeks		119 (27%)	
28^+0–6^ weeks		144 (32%)	
Birthweight	446		950 (780 to 1130)
Twin/triplet	446	115 (26%) / 9 (2%)	
Boys	446	261 (59%)	
Outborn, yes	446	22 (5%)	
Antenatal corticosteroids^a^	440		
None		30 (7%)	
Incomplete		143 (32%)	
Complete		267 (60%)	
Antenatal magnesium sulfate, yes	440	320 (73%)	
Apgar score 5 min	446		8 (7 to 9)
SGA^b^, yes	446	33 (7%)	

^a^Incomplete = birth < 24 h after first dose of betamethasone or last dose of betamethasone administered ≥ 7 days before birth, complete = birth after second dose of betamethasone and last dose of betamethasone administered < 7 days before birth

^b^ < 10th percentile and LGA > 90th percentile according to Fenton 2013 charts [[16]]

### Part A – quantification of stress exposure during NICU admission

**Fig. 2:**
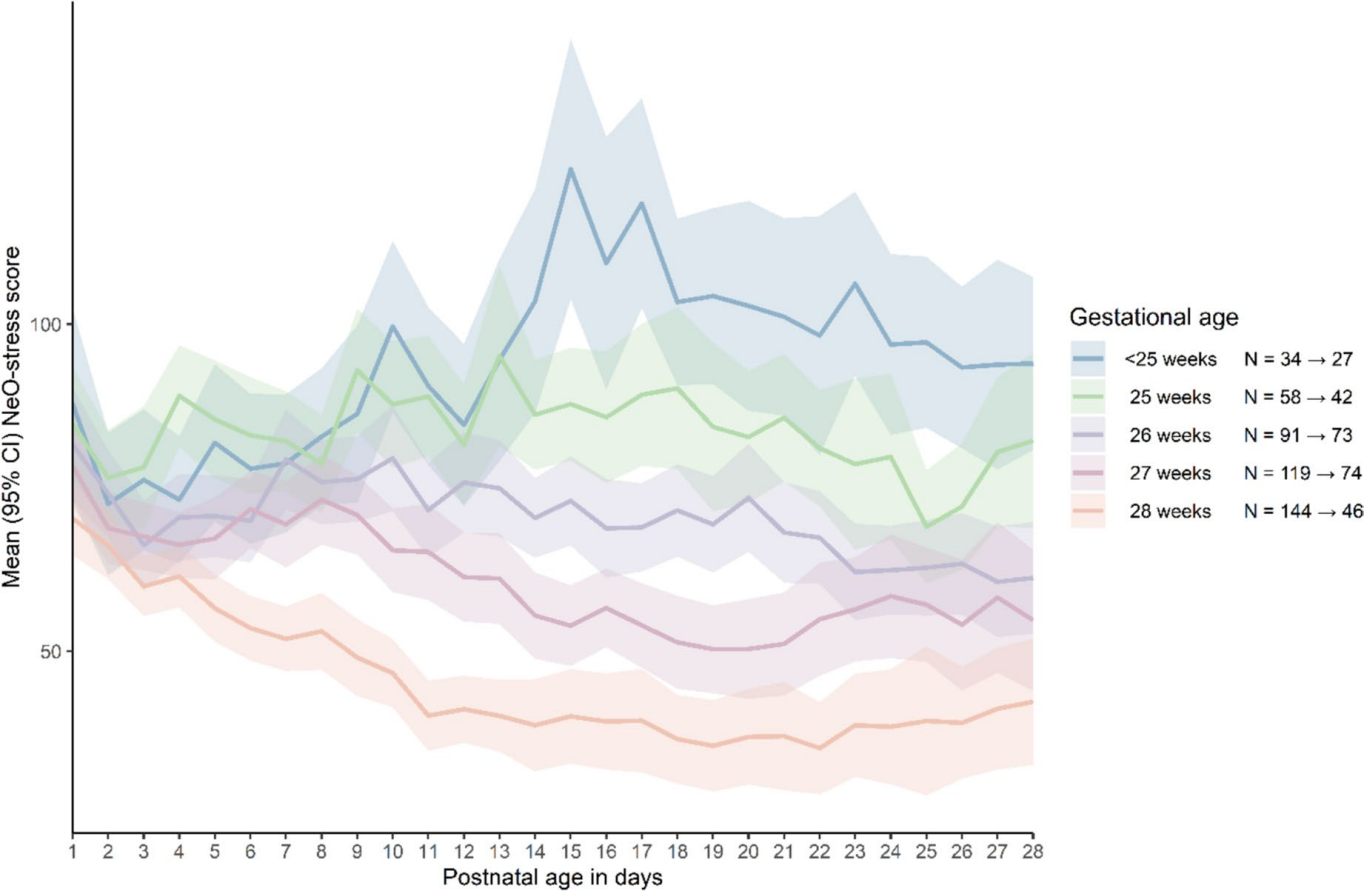
Mean NeO-stress scores (and 95% *CI*) per gestational age group for the first 28 days of life

NeO-stress scores per day ranged from 0 to 317 with a median of 61 (*IQR* 40 to 87). Stress exposure was highest on the day of admission with a median score of 74 (*IQR* 52 to 101). Figure 2 presents the mean NeO-stress scores including 95% *CI* during the first 28 days of life for different gestational age groups.

Table 2 shows a complete overview of the contribution of each stressor (*N*_item_ × *SI*_item_) to the total cumulative NeO-stress score. The stressors which contributed most to the total NeO-stress score were suctioning of nose and mouth (37%) together with endotracheal suctioning (14%). Preterm infants were suctioned (either endotracheal or via nose/mouth) a median of 3.8 times per day (*IQR* 1.7 to 6.7).

**Table 2:** NeO-stress score including contribution per item

*Stressor*	*SI*	%^a^
1 Suctioning of nose and mouth	7	37.3%
2 Endotracheal suctioning	7	13.8%
3 Insertion of a CPAP system with prongs and mask	6	13.7%
4 Insertion of a CPAP system with binasal prongs	6.5	6.5%
5 Non-invasive respiratory support for 24 h	6	5.9%
6 Heel pricks	6.5	5.6%
7 Removing infant from incubator/bed (unwrapped)	5	3.2%
8 Insertion of peripheral venous catheter	7	2.7%
9 Insertion of nasogastric/duodenal tube	6	1.4%
10 Conventional ventilation for 24 h	6	1.2%
11 High frequency oscillation for 24 h	6	0.80%
12 Enema/rectal cannula	5	0.77%
13 Intubation	8	0.67%
14 Insertion of percutaneous long line/central venous line	7	0.50%
15 Venepuncture	7	0.49%
16 Local infection	7	0.48%
17 Having a systemic infection	8	0.47%
18 Insertion of peripheral arterial catheter	7	0.39%
19 Wound care	7	0.38%
20 Surfactant administration via MIST procedure	8	0.35%
21 Umbilical venous/arterial line placement	6	0.33%
22 Extubation	6	0.29%
23 Intramuscular injection	7	0.25%
24 Colostomy care	6	0.25%
25 Surfactant administration via endotracheal tube	7	0.20%
26 Insertion of urinary catheter	6	0.16%
27 Lumbar puncture	8	0.15%
28 Transportation to another hospital	6	0.10%
29 Pneumothorax chest drain in place for 24 h	7	0.08%
30 Ventricular puncture (Rickham or Ommaya)	5	0.07%
31 Application of EEG electrodes (needles)	7	0.07%
32 Surgery	9	0.06%
33 Removal of EEG electrodes	6	0.04%
34 CT scan or MRI	6	0.03%
35 Subcutaneous injection	7	0.02%
36 Insertion pneumothorax chest drain	9	0.02%
37 Removal of chest/wound drain	7	0.01%
38 Application of EEG electrodes (cups)	6	0.01%

^a^This percentage represents the contribution to the total stress exposure, calculated as follows: (*N*_item_ × *SI*_item_) / total cumulative NeO-stress score

### Part B — Factors associated with the level of stress exposure

#### B.1 Infant characteristics associated with the level of stress exposure

A. Linear mixed modelling (see Table 3) including the 411 infants (92%) with complete cases revealed that gestational age and postnatal age are strongly inversely correlated with daily NeO-stress scores with a *p* value of < 0.001 (Model 1). Among the 35 infants with missing data, more infants were outborn, namely 8 (23%) excluded infants compared to 14 out of 411 (3%) included infants.

**Table 3:** Linear mixed modelling with daily NeO-stress score as outcome variable

Outcome	Model 1 *Main effects*			Model 2 *Main effects & interaction effect*			Model 3 *Unit differences*			Model 4 *Open bay vs. single bed*		
**NeO-stress score**	***B***	**95% *CI***	***p*-Value**	***B***	**95% *CI***	***p*-Value**	***B***	**95% *CI***	***p*-Value**	***B***	**95% *CI***	***p*-Value**
Intercept	112.0	100.3 to 123.6	< 0.001**	99.0	87.1 to 110.9	< 0.001**	92.8	81.1 to 104.6	< 0.001**	100.2	88.2 to 112.2	< 0.001**
Postnatal age (PNA; days)	− 0.9	− 1.1 to − 0.8	< 0.001**	0.98	0.56 to 1.4	< 0.001**	1.0	0.57 to 1.42	< 0.001**	0.98	0.56 to 1.4	< 0.001**
Gestational age (GA; days)	− 1.3	− 1.5 to − 1.1	< 0.001**	− 0.69	− 0.94 to − 0.44	< 0.001**	− 0.56	− 0.79 to − 0.34	< 0.001**	− 0.69	− 0.95 to − 0.44	< 0.001**
Boys	0.5	− 3.6 to 4.5	0.82	0.49	− 3.5 to 4.5	0.81	1.8	− 1.7 to 5.3	0.31	0.58	− 3.4 to 4.6	0.78
Antenatal steroids												
No	13.5	3.5 to 23.5	0.008*	13.2	3.2 to 23.1	0.010*	7.4	− 1.4 to 16.2	0.10	12.5	2.5 to 22.4	0.01*
Incomplete	− 0.4	− 4.7 to 3.9	0.86	− 0.30	− 4.6 to 4.0	0.89	− 1.3	− 5.1 to 2.6	0.52	− 0.71	− 5.0 to 3.6	0.75
Complete	REF			REF			REF					
Antenatal MgSO_4_; yes	1.2	− 3.5 to 5.8	0.63	1.1	− 3.5 to 5.8	0.63	0.44	− 3.8 to 4.7	0.84	1.2	− 3.4 to 5.9	0.61
Maternal smoking; yes	− 0.8	− 8.3 to 6.6	0.82	− 0.77	− 8.2 to 6.7	0.84	− 1.8	− 8.4 to 4.8	0.59	− 1.0	− 8.5 to 6.5	0.79
Outborn; yes	9.0	− 2.3 to 20.4	0.12	9.1	− 2.2 to 20.4	0.12	6.2	− 3.7 to 16.0	0.22	9.1	− 2.2 to 20.4	0.11
SGA; yes	5.3	− 1.5 to 12.1	0.12	5.6	− 1.2 to 12.4	0.10	6.4	0.41 to 12.3	0.04*	5.9	− 0.83 to 12.7	0.09
5 − min Apgar score	− 1.6	− 2.9 to − 0.2	0.03*	− 1.6	− 3.0 to − 0.25	0.02*	− 1.8	− 3.0 to − 0.55	0.004*	− 1.6	− 3.0 to − 0.29	0.02*
Site of admission												
NICU 1							REF					
NICU 2							6.5	0.08 to 13.0	0.047*			
NICU 3							− 8.2	− 15.8 to − 0.54	0.04*			
NICU 4							− 5.6	− 13.0 to 1.8	0.13			
NICU 5							9.6	1.6 to 17.6	0.02*			
NICU 6							12.2	4.0 to 20.5	0.004*			
NICU 7							30.5	21.5 to 39.4	< 0.001**			
NICU 8							23.9	15.9 to 31.9	< 0.001**			
NICU 9							− 7.0	− 15.1 to 1.2	0.09			
NICU 10							0.39	− 7.2 to 7.9	0.92			
Unit type												
Single-room unit										− 4.6	− 9.7 to 0.53	0.08
Open bay unit										REF		
Interaction effect												
PNA × GA				− 0.09	− 0.10 to − 0.07	< 0.001**	− 0.09	− 0.11 to − 0.07	< 0.001**	− 0.09	− 0.10 to − 0.07	< 0.001**

This table describes the 4 models used to analyse the effect of the infant and maternal characteristics that were determined in advance together with the interaction between gestational age and postnatal age (models 2, 3 and 4), the NICU of admission (model 3) and the lay-out of the unit (model 4) on the NeO-stress score per patient per day

*REF* reference

^**^ *p* < 0.001, * *p* < 0.05

b. Model 2 shows, however, that the interaction between gestational age and postnatal age significantly affects the NeO-stress score (*B* = − 0.09, 95% *CI* − 0.10–0.06, *p* < 0.001): infants with higher gestational age experience a stronger decline of NeO-stress scores during NICU admission than those with lower gestational age. This model predicts that the NeO-stress score decreases over time for infants with a gestational age above 25^+3^ weeks but actually increases during NICU admission for the younger infants up to 0.9 points per day.

Moreover, infants in which the mothers did not receive antenatal corticosteroid therapy had significantly higher stress exposure with a mean of 12.9 points per day compared to infants from mothers with a complete course of corticosteroids (*B* = 12.9, 95% *CI* 3.2–22.5, *p* = 0.009). A lower 5-min Apgar score was significantly related to higher NeO-stress scores, with each additional point associated with a 1.6 points lower NeO-stress score per day (*B* = − 1.6, 95% *CI* − 3.0–0.27, *p* = 0.02).

#### B.2 Differences in stress exposure between NICUs

The differences between NICUs in models 3 and 4 are corrected for the infant and maternal characteristics included in models 1 and 2. The infant characteristics of the 411 analysed infants are presented per NICU in Table 4.

**Table 4:** Infant characteristics per NICU (*N* = 411)

Variable	*n* (%)/Median (*IQR*)									
NICU	1 *N* = 46	2 *N* = 93	3 *N* = 40	4 *N* = 42	5 *N* = 32	6 *N* = 28	7 *N* = 23	8 *N* = 33	9 *N* = 31	10 *N* = 43
Gestational age (weeks^+days^)	27^+3^ (26^+4^–28^+2^)	27^+3^ (25^+4^–28^+2^)	27^+6^ (26^+5^–28^+3^)	27^+3^ (26^+2^–28^+2^)	27^+1^ (26^+2^–28^+2^)	27^+3^ (25^+6^–28^+5^)	26^+6^ (25^+5^–27^+5^)	27^+0^ (26^+4^–27^+6^)	27^+6^ (26^+4^–28^+3^)	27^+3^ (26^+4^–28^+1^)
Birthweight	1025 (854–1180)	935 (760–1105	998 (888–1123)	950 (800–1130	875 (735–1025)	928 (815–1040)	844 (760–1180)	1000 (830–1090)	980 (780–1200)	945 (735–1215)
Twin/triplet	13 (28%)	27 (29%)	6 (15%)	18 (43%)	6 (19%)	9 (32%)	8 (35%)	7 (21%)	7 (23%)	12 (28%)
Boys	28 (61%)	46 (50%)	27 (68%)	25 (60%)	19 (59%)	17 (61%)	14 (61%)	18 (55%)	20 (65%)	29 (67%)
Antenatal corticosteroids^a^										
None	5 (11%)	1 (1%)	1 (3%)	0 (0%)	1 (3%)	1 (4%)	5 (22%)	2 (6%)		
Incomplete	8 (17%)	46 (50%)	13 (33%)	7 (17%)	10 (31%)	7 (25%)	6 (26%)	10 (30%)		
Complete	33 (72%)	46 (50%)	27 (65%)	35 (83%)	21 (66%)	20 (71%)	12 (52%)	21 (64%)		
Antenatal MgSO4; yes	31 (67%)	79 (85%)	35 (88%)	32 (76%)	26 (81%)	19 (68)	14 (61%)	28 (85%)	23 (74%)	17 (40%)
Maternal smoking; yes	1 (2%)	8 (9%)	6 (15%)	1 (2%)	3 (9%)	0 (0%)	4 (17%)	3 (9%)	3 (10%)	2 (5%)
Outborn; yes	1 (2%)	2 (2%)	1 (3%)	0 (0%)	1 (3%)	1 (4%)	2 (9%)	2 (6%)	2 (7%)	2 (5%)
SGA^b^; yes	3 (7%)	9 (10%)	2 (5%)	6 (14%)	3 (9%)	0 (0%)	1 (4%)	4 (12%)	3 (10%)	7 (16%)
Apgar score 5 min	8 (7–9)	8 (7–9)	7 (6–8	8 (7–9)	8 (6–8)	8 (7–9)	7 (6–8)	8 (7–10)	8 (8–9)	8 (6–8)

^a^Incomplete = birth < 24 h after first dose of betamethasone or last dose of betamethasone administered ≥ 7 days before birth, complete = birth after second dose of betamethasone and last dose of betamethasone administered < 7 days before birth

^b^ < 10th percentile and LGA > 90th percentile according to Fenton 2013 charts [16]

Model 3 shows significant differences depending on the unit of admission (*p* < 0.001, Table 3). Compared to NICU 1 (reference unit), NeO-stress scores per day were on average between 8 points lower (NICU 3) and 30 points higher (NICU 7). The NeO-stress scores for infants born small for gestational age were also significantly higher compared with infants that were appropriate or large for gestational age (*B* = 6.7, 95% CI 0.75–12.6, *p* = 0.006).

Model 4 showed that there was no significant difference in NeO-stress scores between a single bed unit (NICU 4&5, *N* = 74, 18%) compared to an open bay unit (*B* = − 4.5, 95% *CI* − 9.6–0.6, *p* = 0.08), see Table 3.

## Discussion

This multicentre study across all Dutch NICUs in the Netherlands reveals that stress exposure, measured by the NeO-stress score, significantly decreased over time in preterm infants (gestational age 23^+6^ to 29 weeks), though it remained high or increased in those with the lowest gestational ages. Gestational age significantly influenced daily stress levels, consistent with previous findings [17, 18]. This highlights the vulnerability of extreme preterm infants and the need for stress protection. The highest stress exposure on the day of birth might be an underestimation because the day of birth may be less than 24 h. This finding emphasizes that protection against stress should start immediately after birth. Stress exposure unexpectedly increased during the first 28 days of life in the most premature infants, contrasting with previous studies [7, 17]. This likely reflects the stressful journey to clinical stability.

Endotracheal and oral/nasal suctioning were significant contributors to daily stress, highlighting the need to evaluate current suctioning practices. The American Association for Respiratory Care recently developed a clinical practice guideline on endotracheal suctioning of neonatal, pediatric and adult patients [19]. Though suctioning is already performed as-needed in the participating NICUs, this need is based on the expert opinion of the health caregivers and therefore subjective. Airway resistance could guide in determining the need for suctioning [19]. The guideline prescribes keeping suction pressure below − 120 mmHg in neonates and avoiding routine saline irrigation solution. Together with providing facilitated tucking this might lower the stress response [20].

A higher 5-min Apgar score and antenatal corticosteroid administration were associated with lower stress exposure, consistent with Van Dokkum et al.‘s findings in 45 preterm infants [17]. Antenatal corticosteroids may reduce respiratory-related stressors like mechanical ventilation. Both a low Apgar score and lack of antenatal corticosteroid administration are linked to higher mortality risk in preterm infants, with stress exposure potentially mediating this relationship [21–23].

Stress exposure significantly varied across Dutch NICUs, emphasizing the need for benchmarking to identify strategies for stress reduction. Important infant characteristics at birth are included in our model. We realize, however, that NICUs might differ in other factors, such as access to surgery and parents’ socioeconomic status [24]. Previous studies have shown differences between NICUs regarding pain assessment and analgosedation [25–27], but this is the first study to analyse stress exposure. Stress exposure was not significantly different between open-bay and single-bed NICUs. While single family rooms are known to improve parental presence, involvement and skin-to-skin care [28], which can reduce stress levels, the impact of unit type on stress response was beyond this study’s scope. These findings provide a foundation for comparing clinical practices and benchmarking across centres.

Our insights in clinical stressors provide NICU professionals with opportunities to decrease stress exposure. We should first aim to decrease the frequency of the clinical stressors. Evidence on how to achieve this decrease is currently lacking and it is assumed that this ability might be limited [2, 29]. Focusing on the procedure itself (e.g. providing containment) is therefore also important to minimize the stress effect [30]. Reducing both the frequency and the stressfulness of each clinical stressor, resulting in a lower Severity Index, will optimally decrease stress exposure. Parents also play a crucial role, with practices like skin-to-skin care [11, 31]. The European Standards of Care for Newborn Health advocate for initiating and maintaining skin-to-skin care as early as possible [32].

A major strength of this study is the quantification of stress exposure in a large national cohort of 446 preterm infants. However, limitations include focusing on clinical stressors during NICU admission and using a fixed severity index, which may not reflect variations due to stress-reducing interventions or individual infant conditions. Additionally, prospective nurse-reported data carries a risk of under-registration.

Focusing on clinical stressors is a key step in minimizing stress, as they initiate the infant’s response [10]. The next steps are to put further focus on the other elements of the stress framework as proposed by Lu et al., such as the stress response and stress effect, in order to better understand the way stress thereafter impacts the infant’s development [10]. Examining other NICU-related stressors, such as light, noise and limited parental presence will further identify ways to reduce stress exposure. Additionally, assessing the role of interventions, both non-pharmacological and analgosedation, will help clarify stress consequences on development. To assess these consequences on development, we currently collect neurodevelopmental outcome data including motor, cognitive and language development, as well as data on parental wellbeing.

The ultimate goal would be to minimize the negative consequences of stress and improve quality of life for all preterm born children and their families [33]. Protective quality improvement programs should include evidence-based interventions that help decrease either the frequency or the stressfulness of clinical stressors. Proven stress-reducing interventions, such as skin-to-skin care, should be offered to all (preterm) infants [34, 35]. Other interventions might be either considered not feasible to provide continuously to all preterm NICU infants, e.g. music therapy [36] or even harmful, e.g. routine use of analgosedatives [37, 38]. Risk profiles based on our findings can guide individualized, effective pharmacological and non-pharmacological strategies for each infant and family.

## Conclusion

The youngest, most vulnerable infants face the highest cumulative stress during NICU admission. Stress prevention should begin immediately after birth and be maintained throughout their stay. Lower 5-min Apgar score and lack of antenatal corticosteroid administration can help identify the infants at greater risk of high stress exposure at birth. Understanding the extent of daily stress exposure, as well as the variations across individuals and hospitals, offers valuable opportunities to decrease stress exposure and identify high-risk infants. Prioritizing stress-reduction programs is essential to safeguard preterm infants from the negative effects of early life stress.

## Data Availability

The data that support the findings of this study are available from the corresponding author, S.S., upon reasonable request.

## Abbreviations

CPAP: Continuous positive airway pressure
MgSO_4_: Magnesium sulfate
NICU: Neonatal intensive care unit
*SI*: Stressfulness index
